# Surgery of Lymphatic Glands

**Published:** 1905-12-30

**Authors:** 


### SURGERY OF LYMPHATIC GLANDS.

**On the Removal of Tuberculous Lymph Glands from the Neck.**—Although this is one of the commonest operations in surgery, there is great need for emphasising the advisability of making the incisions in such a manner that the resulting scars will be as inconspicuous as possible. Probably the majority of such operations are still performed through an incision along the anterior border of the sterno-mastoid muscle. But in nearly every case the scar of this incision becomes stretched, or thickened by keloid, and, situated as it is, a very unsightly and conspicuous cicatrix is the result. Dowd[1] makes the principal incision to skirt the sub-maxillary triangle, and Sutcliffe[2] suggests practically the same as his chief skin cut. It follows the line of the digastric muscle from the mastoid process down to the hyoid bone, and then up to the jaw just below and outside the symphysis. The scar of this lies in the natural fold between the lower jaw and the neck. It lies over the most common site for enlarged tubercular glands, namely, the sub-maxillary and superior carotid sets. Dowd points out that the lowest fibres of the cervico-facial division of the seventh nerve are exposed by this incision, as they lie just below the ramus of the jaw. This nerve should be found and preserved, otherwise the depressor labii inferioris will be paralysed. In order to reach the glands which lie below the lower two-thirds of the sterno-mastoid muscle, and also those in the posterior triangle of the neck, it is better to make a second incision rather than to extend the first. This may either run along the posterior border of the sterno-mastoid or, beginning at the hair margin over the anterior border of the trapezius, come downwards and forwards across the lower part of the posterior triangle. However the posterior and deep glands are exposed, the first care should be to dissect out and preserve the spinal accessory nerve, the injury to which is the commonest accident attending these operations. It is a matter of opinion whether it is ever necessary to cut the sterno-mastoid muscle or to deliberately tie the internal jugular vein. Where a large mass of glands lies over the carotid sheath at the root of the neck, section of the muscle will make the operation much easier and will involve less risk than burrowing under it. The adhesion of the glands to the deep vein is seldom very close, and when it has to be tied it is usually because of accidental wounding. In reviewing 100 cases on which he had operated, Dowd comes to the following conclusions: that tuberculous glands of the neck are due in 86 per cent. to infection through fauces, pharynx, or nose; that 75 per cent. are free from recurrence, and that the immediate mortality of the operation is nil; that in cases not operated on about 25 to 50 per cent. develop tuberculosis of the lungs or other internal organs.

Corner and Dudgeon[3] describe a condition of acute tuberculous infection of the wound area after the removal of tubercular glands, which, according to their experience, is not very uncommon, as they can bring forward upwards of six cases of recent occurrence. In such cases swelling occurs in the area of the scar after the removal of the glands. This may become quite as large as the original tubercular mass. It is painful and somewhat inflamed, and generally remains stationary unless it is operated on. But in some cases it undergoes spontaneous resolution. When the swelling is opened up all the tissues which were left apparently healthy at the original operation are found to be grey and infiltrated by tubercular material, and microscopic examination confirms this and shows giant cell-systems and typical "tubercles" in the muscle and connective tissue.

When all the affected tissues have been scraped out and the cavity loosely packed, rapid healing takes place. And the authors conclude, therefore, that temporary drainage should be employed in all these operations on tubercular tissues.

[1] Annals of Surg., July, 1905. [2] Brit. Med. Jour., May 13, 1905. [3] Clin. Jour., July 12, 1905.

